# The role of inflammatory serum markers and ureteral wall thickness on spontaneous passage of ureteral stone < 10 mm: A prospective cohort study

**DOI:** 10.1016/j.amsu.2022.104198

**Published:** 2022-07-16

**Authors:** Ismaeel Aghaways, Rebaz Ibrahim, Rawa Bapir, Rawezh Q. Salih, Karzan M. Salih, Berwn A. Abdulla

**Affiliations:** a Department of Surgery, College of Medicine, University of Sulaymaniyah, Sulaymaniyah, Iraq; b Department of Urology, Sulaymaniyah Surgical Teaching Hospital, Sulaymaniyah, Iraq; c Smart Health Tower, Madam Mitterrand Street, Sulaymaniyah, Iraq; d U-merge Ltd. (Urology in Emerging Countries), London, UK; e Kscien Organization, Hamdi Str, Azadi Mall, Sulaymaniyah, Kurdistan, Iraq; f Iraqi Board for Medical Specialties, Department of Surgery, Sulaymaniyah Center, Sulaymaniyah, Iraq

**Keywords:** Medical expulsive therapy, Urolithiasis, Ureteral stone passage, Inflammatory serum markers, Ureteral wall thickness

## Abstract

**Introduction:**

Ureteral stone is a worldwide disease and accounts for 20% of all urolithiasis. There is a widespread discussion on the preferred initial treatment method, whether medical or surgical, and each has its pros and cons. In this study, we aimed to assess the role of both ureteral wall thickness around the stone and inflammatory markers in guiding the decision-making process.

**Methods:**

In this prospective study, 161 patients who presented with ureteric colic and were diagnosed with ureteral stone with NCCT were included. UWT around the stone was measured, and the NLR and PLR were calculated. The patients were given a single daily dose of tamsulosin 0.4 mg for 4 weeks with weekly follow-up to determine SSP or failure.

**Results:**

Of the 161 patients with a mean age 40.12 ± 12.36 SD, 55.9% had a spontaneous stone passage. Receiver operating characteristics showed a cut off value of 2.45 mm UWT of non SSP patients with an 83% sensitivity and 86% specificity. Moreover, there was a significant correlation between higher NLR, PLR and increased UWT (Pearson correlation of 0.314 and 0.426 respectively). The combined higher NLR, PLR and increased UWT were associated with failure of SSP (p-value <0.001).

**Conclusion:**

Many factors play a role in decision making for management of ureteral stones. Our study concludes that patients with high NLR, PLR, and UWT around the stone have lesser chance of SSP using MET. Their rise can be used as predictors to decide early intervention.

## Highlights

• Ureteral stone is a worldwide disease and accounts for 20% of all urolithiasis.
• Impacted ureteral stones occupy the majority of emergency department visits due to urolithiasis.
• The role of inflammatory serum markers and UWT around the stone on spontaneous passage are controversial.

## 1 Introduction

Urolithiasis is a worldwide common disease with an incidence as high as 20%. The prevalence of ureteral stones has increased over the past years, and it constitutes 20% of all urolithiasis [1,2]. Impacted ureteral stones occupy the majority of emergency department visits due to urolithiasis and causes a high economic burden to the health system [3]. This burden together with the rising incidence of ureteral stones, encourages a vigilant treatment plan, including medical expulsive therapy and timing of intervention [4]. Surgical intervention for ureteral stones could be regarded as overtreatment and adds extra costs because guidelines state that patients with uncomplicated ureteral stones smaller than 10 mm can be offered conservative management, including watchful waiting or medical expulsive therapy for 4 weeks. On the other hand, watchful waiting could have undesirable outcomes like urosepsis and renal impairment [[5], [6], [7]]. However, it is controversial which patient would likely benefit from conservative management or immediate intervention. For that reason, it is imperative to identify the factors predicting spontaneous ureteral stone passage or stone-related complications during the period of conservative treatment [8]. Distally located small-sized stones in the ureter have been found in most studies as a predictor of spontaneous stone passage [9]. Identifying ureteral wall thickness around the stone using CT scan and raised inflammatory serum marker, which could reflect the inflammation and impaction of the stone in the ureter, have also been investigated as parameters to predict SSP. However, their role is controversial [[10], [11], [12], [13], [14], [15]]. Some authors suggested that high leucocyte count is an indicator for stone passage, hypothesizing that stone passage causes ureteral wall inflammation in comparison to the static one [12]. On the contrary, other investigators found a high leucocyte count in correspondence to a low rate of spontaneous ureteral stone passages or even early intervention during MET [13,14]. Furthermore, a more recent study did not show a significant association between spontaneous ureteral stone passage and WBC count [15.16]. In the present study, we aimed to assess the role of both ureteral wall thickness around the stone and inflammatory serum markers in predicting SSP of <10 mm ureteral stones.

## 2 Methods

### 2.1 Registration

The current study was registered in accordance with the Helsinki declaration – “Every research study involving human subjects must be registered in a publicly accessible database before recruitment of the first subject”. The study was registered in the Research Registry with a registration number of (8072). The link ishttps://www.researchregistry.com/register-now#home/registrationdetails/62c2c16dda82dc001e5989dd/

### 2.2 Setting and study design

A prospective analysis was performed for 176 patients from January 2021 to December 2021 who presented with acute ureteric colic to the emergency unit. It was written in line with STROCSS 2021 guidelines [17].

### 2.3 Ethical considerations

The study was performed following the approval of the Ethical Committee from the Kurdistan Board for medical specialties (No 1135). Consent was taken from all participants.

### 2.4 Inclusion and exclusion criteria

Patients with a radiological diagnosis of a ureteric stone of ≤10 mm on a non–contrast-enhanced Computerized Tomography (NCCT), willing to receive medical expulsive therapy and further follow up, were included in the study. However, patients with multiple ureteral stones, concurrent renal stones, solitary kidneys, associated chronic inflammatory conditions, taking, positive urine culture, high fever on presentation, renal impairment, and patients who were lost in the follow-up were excluded from the study. Based on that, 15 participants were excluded, and the data of only 161 patients were analyzed.

### 2.5 Data collection

Demographic data such as age, gender, and body mass index (BMI) were collected. A medical history of diabetes mellitus and hypertension, as well as a history of previous ureteral stone passages were obtained.

### 2.6 Diagnostic assessment

During the acute phase, urine and blood samples were collected, and their results with radiographic examinations were recorded. The neutrophil-to-lymphocyte ratio (NLR) and platelet-to-lymphocyte ratio (PLR) were then calculated as inflammatory markers.

The NCCT provided site (proximal, mid and distal), size (was defined by the stone's largest diameter), density (Hounsfield unit), degree of hydronephrosis (no, mild, moderate and severe using Onen classification [18]) and ureteral wall thickness (UWT) around the stone.

Axial NCCT images of 5 mm thickness slices with soft-tissue radiodensity (a window width of 360, a pitch of 1.5, a tube voltage of 120 kV and a tube current of 70–90 mAs were the setting parameters) were used to evaluate the stone and its surrounding tissue. The UWT was calculated by locating the point with the greatest soft-tissue thickness (ureteral wall ± peri-ureteral edema) around the circumference of the stone.

### 2.7 Management procedure

The patients were put on a daily single dose of tamsulosin 0.4 mg for four weeks with a regular weekly interval visit to check for spontaneous ureteral stone passage or stone-related complications. Failure of passage was defined as the presence of the stone on NCCT at the end of the 4 weeks or urgent intervention by drainage, shockwave lithotripsy, or URS due to stone-related complications within the period.

### 2.8 Statistical analysis

Data were analyzed using the Statistical Package for the social sciences, version 21.0 (IBM corporation, Armonk, NY). The p-value of <0.05 was regarded as significant. The clinical variables were compared using Chi-square or an independent *t*-test. Univariate and multivariate analysis was performed and the Odds ratio was calculated to assess the association of variables with the stone passage.

Receiver operator curve (ROC) analysis for the area under the curve (AUC) values was done to derive the cut-off values for UWT in non-spontaneous stone passage patients. Pearson correlation was performed for the association of UWT with both PLR and NLR. Manova test was used for the relation between NLR, PLR, and ureteral wall thickness for ureteral stone passage.

## 3 Results

**Fig. 1:**
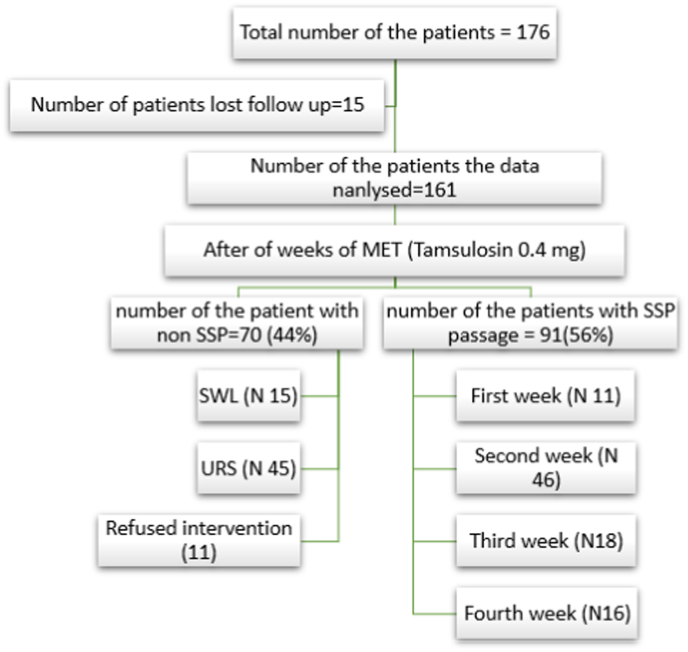
Follow up flow chart of the participants.

Of the 176 cases that fulfilled the inclusion criteria, 15 patients were excluded due to loss of follow-up. The mean age of the patients was 40.12 ± 12.36 SD, with the range being (17–78). Eighty-seven patients (54%) were female, and 74 patients (46%) were males. Fig. 1 shows the follow-up flow chart of the patients. The comparison of demographic data, patients, and stones characteristics between the SSP and non-SSP are mentioned in Table 1. Neutrophil, platelet, NLR and PLR were significantly higher in the non SSP patients with a p-value of 0.01, <0.001, 0.01, and <0,001 respectively. The stone size was also significantly larger in non-SSP patients (p value < 0.0001). Moreover, the distally located stones were more likely to spontaneously pass in comparison to proximal stones with a p-value of 0.019.

**Table 1:** Comparison of demographic, laboratory, and radiological factors as predictors of spontaneous stone passage.

Variable	Total	No SSP	SSP	p.value
Age(Year±SD)	40.12 ± 12.36	42.27 ± 13.87	38.43 ± 10.81	0.050*
Gender				
Male (n,%)	86(53.5)	41(57.7)	45(50)	0.328**
Female (n,%)	75(46.5)	30(42.3)	45(50)	
BMI(Kg/m^2^)	22.47 ± 2.4	22.6 ± 2.35	22.29 ± 2.59	0.309*
Diabetic				
Yes (n,%)	19(11.8)	4(5.6)	11(12.7)	0.153**
No (n,%)	142(88.2)	67(9.4)	79(87.7)	
Hypertension				
Yes (n,%)	19(11.8)	8(11.3)	11(12.3)	0.852**
No (n,%)	142(88.2)	63(88.7)	79(87.8)	
WBC(mean ± SD)	9462 ± 1861	9710 ± 1650	9267 ± 2001	0.136*
PLT mean ± SD)	272.33 ± 48.6	293.9 ± 40.13	255.3 ± 48.1	<0.001*
Neutrophil (%)	63.59 ± 9.21	66.3 ± 7.7	61.4 ± 9.7	0.01*
PLR (mean ± SD)	10.09 ± 3.8	11.47 ± 4.86	9.00 ± 2.30	<0.001*
NLR (mean ± SD)	2.39 ± 1.05	2.63 ± 1.35	2.20 ± 0.69	0.010*
Serum creatinine	0.76 ± 0.16	0.74 ± 0.15	0.78 ± 0.17	0.109*
Stone size(mean ± SD)	7.19 ± 1.68	7.76 ± 1.49	6.43 ± 1.60	<0.001*
Stone location				
Upper(n,%)	8(5)	6(8.5)	2(2.2)	
Mid(n,%)	24(15)	15(21)	9(10)	0.019**
Lower (n,%)	129(80)	50(70.4)	79(87.8)	
HU	710	726 ± 218	697 ± 215	0.403*
UWT	2.42 ± 0.73	2.98 ± 0.67	1.98 ± 0.42	<0.001*
HN				
No mild	109(67.7)	40(36.6)	69(63.4)	0.006**
Moderate-severe	52(32.3)	31(59.6)	21(40.4)	

BMI body mass index, WBC white blood cells, PLT platelet, PLR platelet lymphocyte ratio, NLR neutrophil lymphocyte ratio, UWT ureteral wall thickness, HU Hounsfield unit, SSP spontaneous stone passage, * independent *t-*test, ** chi-square test.

Our results showed that the younger patients were more likely to pass the stones than the older ages.

Concerning the grade of hydronephrosis, there was a significantly higher rate of stone passage in patients with no or mild hydronephrosis compared to moderate to severe hydronephrosis (p-value = 0.006). However, on multivariate analysis the association was not significant (p-value 0.051).

**Fig. 2:**
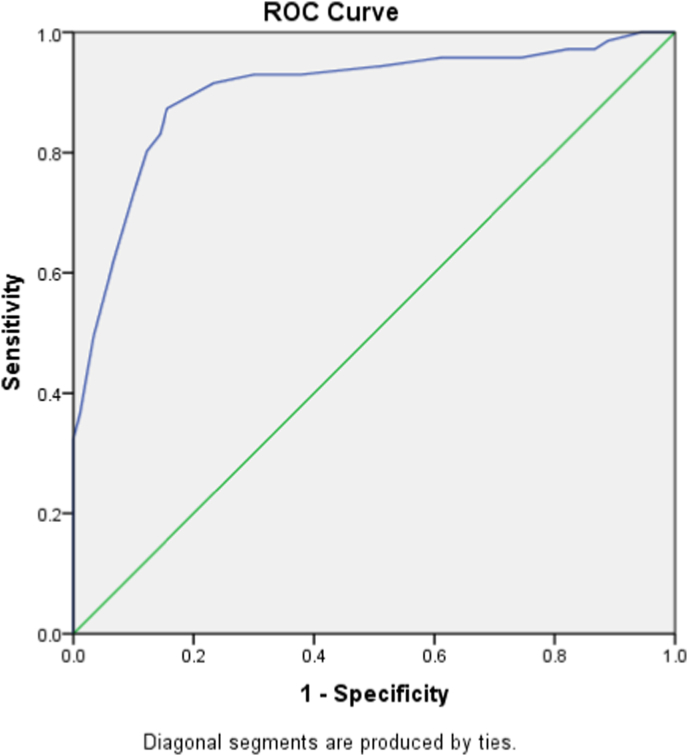
Receiver operating characteristic curve for ureteral wall thickness and non-spontaneous stone passage. Area 0.901 (95% CI 0.849–0.952) with a cut-off value of 2.45 mm of UWT with an 83% sensitivity and 86% specificity.

Ureteral wall thickness around the stone was another factor that significantly affected ureteral stone passage. Our result showed that lower ureteral wall thickness was associated with a higher chance of stone passage, and the correlation was highly significant (p-value < 0.0001). Receiver operating characteristic curves showed an area of 0.901 (95% CI 0.849–0.952) with a cut-off value of 2.45 mm of UWT with an 83% sensitivity and 86% specificity (Fig. 2). However, several other factors like sex, medical comorbidities, HU of the stone, creatinine level, and hematuria did not affect the outcome.

Univariate and multi multivariate analysis of the factors related to the failure of SSP revealed a significant correlation between higher UWT, NLR, PLR, and stone size with failure of SSP with an OR of 11.45, 3.24, 7.54, and 3.24, respectively (Table 2).

**Table 2:** Univariate and multivariate analysis of the factors associated with failure of spontaneous ureteral stone passage.

	Univariate			Multivariate		
Variable	OR	95% CI	*P*	OR	95% CI	*P*
**UWT**	6.56	5.25–18.24	<0.001	11.45	3.24–25.76	0.002
**NLR**	1.686	1.112–2.557	0.014	3.24	1.65–6.23	<0.001
**PLR**	4.02	1.324–10.43	0.002	7.54	4.23–12.54	0.004
**Age**	1.026	1.00–1.053	0.053			
**Gender**	1.367	0.730–2.557	0.328			
**BMI**	1.060	0.934–1.203	0.368			
**Hydronephrosis**	2.546	1.294–5.012	0.007	4.143	1.765–9.054	0.051
**Stone size**	1.690	1.357–2.104	<0.001	3.25	2.11–8.25	<0.001
**Previous history of ureteral stone**	0.43	0.017–0.564	0.03	1.09	0.71–4.98	0.354

BMI body mass index, WBC white blood cells, PLT platelet, PLR platelet lymphocyte ratio, NLR neutrophil lymphocyte ratio, UWT ureteral wall thickness, HU Hounsfield unit, SSP spontaneous stone passage.

Our results showed a correlation between higher NLR, PLR and UWT (Pearson correlation of 0.314 and 0.426 respectively with a p-value of both <0.001).

Manova test for the relation between PLR, and NLR with UWT for SSP showed a significant chance of SSP toward the lower PLR, and NLR with lower UWT (Table 3).

**Table 3:** Manova test for relationship between NLR and ureteral wall thickness at sone site with Spontaneous stone passage.

Variable	No SSP	SSP	p-value
NLR	2.63 ± 1.35	2.20 ± 0.69	<0.001
UWT	2.98 ± 0.67	1.98 ± 0.42	
PLR	11.47 ± 4.86	9.00 ± 2.30	<0.001
UWT	2.98 ± 0.67	1.98 ± 0.42	

PLR platelet lymphocyte ratio, NLR neutrophil lymphocyte ratio, UWT ureteral wall thickness, SSP spontaneous stone passage.

## 4 Discussion

A variety of management strategies exist to treat ureteral stones, ranging from conservative treatment (medical expulsive therapy) to shock wave lithotripsy to ureteroscopy [19]. Medical treatment is considered non-invasive and cheap; however, it may have undesirable consequences like renal impairment, urinary tract infection, recurrent colic, and patient discomfort. On the other hand, shockwave lithotripsy and endoscopic stone removal are safer and yield better stone-free rates than medical treatment, yet they are costly, and procedure-related complications such as urinary infection, hematoma formation, and urinary extravasation should be kept in mind [20]. Likewise, postponing surgical intervention until failure of medical therapy is stressful to the patient and adds to the cost of treatment when compared with immediate surgical interference [21]. This vagueness in clinical decision-making has prompted many investigators to explore markers to guide clinicians in triaging patients for optimal treatment plans [22].

Different pharmacological agents such as alpha-blockers, calcium channel blockers, phosphodiesterase inhibitors, and corticosteroids are studied to facilitate spontaneous ureteral stone expulsion. However, recent guidelines concluded the superiority of using alpha-blocker monotherapy in this regard [23]. In the current study, our patients received a single dose of 0.4 mg of tamsulosin daily for 4 weeks.

### 4.1 Stone size and location

Stone size and location are among the important factors to predict spontaneous stone passage. Stones < 5 mm in any part of the ureter have a 75% chance of spontaneous passage. As the size increases, the rates of spontaneous passage decline (60% for 5–7 mm, 48% for 7–9 mm, and 25% for > 9 mm). Based on the stone location, the SSP will change ranging from 79%, 60%, and 48% for distal, mid, and proximal ureteral stones respectively. The EAU/AUA panel studied SSP based on a meta-analysis that showed 68% for <5 mm stones and 48% for 5–10 mm stones [24]. The current study showed a 55.9% SSP rate, and the stone size was significantly smaller in comparison with non SSP patients (6.43 ± 1.60 versus 7.76 ± 1.49) with a p-value < 0.001, while the rate of SSP concerning the stone location was 25%, 37,5%, and 61.2% for the proximal, mid and lower ureteral stones respectively which is relatively lower than the previously mentioned studies. The overall lower SSP in our study, is likely related to the higher mean size of the stones in our patients (7.19 ± 1.68 mm).

### 4.2 Inflammatory serum markers and SSP

NLR is regarded as a parameter to evaluate the inflammatory status of a patient. Its prognostic efficacy has been proved in various kinds of cancers, postoperative complications, major cardiac events, and various infectious states, yet its role in predicting SSP is controversial [[25], [26], [27], [28], [29]]. Sfoungaristos et al. found that elevated WBC and neutrophile in patients with spontaneous ureteral passage. They hypothesized that stone passage causes ureteral wall inflammation, and this process does not occur in statis stones [12]. By contrast Nassib et al., in their analysis of 619 patients with ureteric stone <10 mm, observed an elevated NLR and PLR in SSP failure patients with an odds ratio of 2.96 (95% CI 1.80–5.49) for NLR between 2.87 and 4.87, and an OR of 3.28 (1.79–6.19) for PLR between 10.42 and 15.25 [22]. Another study by Kwang Suk Lee revealed a significant correlation between SSP and low NLR, with an odds ratio of 9.3 for NLR of <2.3 [30]. Our results are in line with the mentioned studies. We found a higher NLR and PLR among patients with failure of SSP, with an OR of 3.2 (95% CI 1.65–6.23), P-value <0.001 and an OR of 7.53 (95% CI 4.3–12.54), p-value 0.004 respectively. Nonetheless, Ahmed et al. and Senel C et al. did not find a relation between WBC count, NLR, and PLR with SSP [15,16].

### 4.3 UWT around the stone and SSP

Impacted ureteral stone causes inflammatory reactions that may result in ureteral wall edema, hypertrophy, and fibrosis which ultimately increases the ureteral wall thickness around the stone [31]. Several studies have evaluated the value of ureteral wall thickness in the treatment of ureteral stones. Ozbir et al., Elibol et al., and. Sarica et al. found in their studies that the UWT can be regarded as a predictor of stone impaction [[32], [33], [34]]. Moreover, Yoshida et al. concluded its use as a dependable factor to predict stone impaction and surgical outcome post ureteroscopy [35]. However, its value for predicting spontaneous ureteral stone passage and the outcome of MET is questionable [10,36]. Different cut-off values of UWT have been reported to predict SSP or outcome MET in the literature. Yoshida et al. [10] reported 2.71 mm as a cut-off level predicting SSP in 4 weeks, while Mohamed Samir et al. [11] reported a cut-off level of ≥3.75 mm predicting stone passage failure. The current study showed a cut-off value of 2.45 mm with an 83% sensitivity and 86% specificity, which is comparable with the literature. Additionally, our study revealed a significant positive correlation between NLR and PLR with ureteral wall thickness with a p-value of <0.001 on one hand, and on the other hand, the higher ureteral wall thickness with elevated NLR and PLR was associated with failure of SSP, the relation was statistically significant (p-value < 0.001). To the best of our knowledge, our study is the first of a kind to use both factors at the same time to predict SSP.

There are limitations to our study, starting with the small sample size and the single-center data. Another limitation is missing some important data like C reactive protein and ESR, which are important inflammatory markers that can be used to predict SSP. However, due to the economic burden, we could not send such investigations to all the patients. Another limitation is using a single sample during the patients' presentation rather than repeating it to observe the trend of inflammatory markers is regarded as another drawback to our study.

## 5 Conclusions

Many factors play a role in the decision making of ureteral stone, and it is a complex process. Ureteral stone passage is dependent on the stone size and location. Our findings propose that higher NLR and PLR, together with the higher ureteral wall thickness at the stone site, suggest failure of spontaneous stone passage during MET. However due to vast controversies in the literature, further prospective and comprehensive studies are recommended to confirm our results.

## Ethical approval

The manuscript approved by ethical committee of the Kurdistan Board for medical specialties (No 1135).

## Sources of funding

No source to be stated.

## Author contribution

Ismaeel Aghaways: supervisor the project, final approval of the manuscript.

Rawa Bapir: literature review, writing the first draft of the manuscript, and final approval of the manuscript.

Karzan M. Salih: wrote and an amended the second draft, final approval of the manuscript.

Berwn A. Abdulla, Rawezh Q. Salih,: literature review, final approval of the manuscript.

Rebaz Ibrahim: data collection, follow up of the patient, final approval of the manuscript.

## Registration of research studies

The study was registered in the Research Registry with a registration number of (8072). The link is https://www.researchregistry.com/register-now#home/registrationdetails/62c2c16dda82dc001e5989dd/

## Guarantor

Rawa Bapir is Guarantor of this submission.

## Consent

Consent has been taken from the patients and the family of the patients.

## Provenance and peer review

Not commissioned, externally peer reviewed.

## Declaration of competing interest

There is no conflict to be declared.
